# Manipulation under anesthesia versus physiotherapy treatment in stage two of a frozen shoulder: a study protocol for a randomized controlled trial

**DOI:** 10.1186/s12891-017-1763-2

**Published:** 2017-10-11

**Authors:** Tim Kraal, Bertram The, Ronald Boer, M. P. van den Borne, Koen Koenraadt, Pjotr Goossens, Denise Eygendaal

**Affiliations:** Department of orthopaedic surgery, Amphia ziekenhuis Breda, Molengracht 21, 4818 CK Breda, The Netherlands

**Keywords:** Frozen shoulder, Adhesive capsulitis, Manipulation under anesthesia, Physiotherapy

## Abstract

**Background:**

There is no consensus about the optimal treatment strategy for frozen shoulders (FS). Conservative treatment consisting of intra-articular corticosteroid infiltrations and physiotherapy are considered appropriate for most patients. However, with a conservative strategy, patients experience a prolonged rehabilitation period with a considerable amount of pain and disabilities in daily life. Also, at long term, a residual amount of pain and restriction of range of motion is frequently reported. Manipulation under anesthesia is a short and relative simple procedure with the potential to rapidly reduce symptoms and restore the range of motion. The objective of this trial is to evaluate the effectiveness of MUA followed by a PT program compared to a PT program alone, in the treatment of patients with a stage two FS. We hypothesize that the course of the disease can be shortened with MUA with a quicker functional recovery.

**Methods:**

This is a prospective, single center, randomized controlled trial. Eligible patients will be allocated to either the manipulation (MUA) group or the physiotherapy alone (PT) group. In the MUA group manipulation will be performed under interscalene block, directly followed by an intensive physiotherapy treatment protocol, with the goal to maintain the obtained range of motion. Patients allocated to the PT group are given advice and education and receive a written protocol to hand out to their physical therapist based on the recent guideline of the Dutch Shoulder Network for the treatment of frozen shoulders. Descriptive statistics will be used to describe the sample size, patients demographics, presence of diabetes mellitus, range of motion, duration of symptoms till randomization and will be presented for each treatment group. The SPADI is used as primary functional outcome parameter. Secondary outcome parameters are; OSS, NPRS, EQ-5D 3-L, passive range of motion, WORQ-UP, duration of symptoms, usage of analgesics and adverse events. A sample size of 41 subjects in each group was calculated. Follow up is planned after 1,3 and 12 months. The length of physiotherapy treatment in both groups is variable, depending on individual progression. Differences between groups in outcome parameters will be analysed using the linear mixed modelling and the restricted maximum likelihood ratio technique for estimating the model parameters.

**Discussion:**

Successful completion of this trial will provide evidence on the best treatment strategy for patients with a stage two frozen shoulder. The results of this study can lead to a better understanding for the role of manipulation in the treatment of frozen shoulders.

**Trial registration:**

This trial is registered in the Dutch Trial Register under the number NTR6182 on the 20th of February 2017.

## Background

Frozen shoulder (FS) is a common cause of shoulder pain and disability. It affects approximately 2-4% of the general population [1], with a peak incidence between the fifth and sixth decade. FS is slightly more frequent in women than in men, and the most important associated condition is diabetes [1, 2]. The pathophysiology of idiopathic FS is still poorly understood [3]. Idiopathic FS is characterised by a spontaneous onset of pain and stiffness of the shoulder, especially a loss of external rotation, without a prior traumatic event [4]. FS is traditionally divided in three stages [5]. Stage one is called the “freezing stage” and is characterised by severe pain and increasing stiffness. Stage two is the “frozen stage” with established stiffness and reduced pain at rest, but still painful at the end of the range of motion. In the third stage, the “thawing stage”, gradual improvement of motion occurs. Earlier studies considered it to be a self-limiting, reversible condition [5, 6]. Conservative treatment, most frequently consisting of physiotherapy (PT) and corticosteroid infiltrations, is considered appropriate for the majority of patients [4]. However, with conservative treatment residual pain is reported in up to 50% of patients and measurable restriction of motion in up to 60% [7, 8]. Functional limitations at long term occur in 6 - 16% of patients [9, 10]. Also, natural history studies suggest an average duration of 30.1 months [11]. Patients experience a prolonged rehabilitation period with a considerable amount of pain and disability in daily life. Their functional limitations can lead to absenteeism at work [12–14]. There are several invasive treatment procedures possible, like manipulation under anaesthesia (MUA), arthroscopic capsular release and hydrodilatation. However, good quality comparative studies concerning these procedures are scarce. Systematic reviews point to a lack of evidence, with no consensus about superiority of one of these procedures [11, 15–17].

Traditionally, manipulation under anaesthesia (MUA) is a well-established treatment for FS if conservative treatment fails [13, 18, 19]. MUA is a short and relative simple procedure by which capsular adhesions are torn apart by manipulation, with the potential to rapidly restore the range of motion and reduce symptoms within days after the procedure [20]. However, the role of MUA in the treatment of FS is still controversial because it might lead to serious complications in rare cases such as a humeral fracture, glenohumeral dislocation, and brachial plexus traction injury [21, 22]. Other potential complications are intra-articular damage to the cartilage, glenoid rim fractures, or rotator cuff tears [23]. On the other hand, rotator cuff integrity was maintained after MUA in the study of Atoun [24] and the reported complication rate in cohort studies and reviews of 0.5% is rather low [16, 20, 25].

There is only one randomized controlled trial, in which MUA is compared to conservative treatment. Kivimäki et al. conducted a randomized trial with 110 patients in which MUA in combination with a home exercise program was compared to a home exercise program alone [14]. A small difference regarding mobility and pain in favour of the manipulation group was found, but was considered clinically unimportant. However, 34% of patients were lost to follow up after 6 months, and only 3 patients of the manipulation group were available for follow up at 12 months. Therefore, no firm conclusions can be drawn based on that study. Moreover, the rehabilitation after MUA was far different from the physiotherapy protocol in the current study. In the study of Kivimäki et al., physiotherapy advice was given in two sessions and written instructions for a home exercise program were provided. We suppose that an initial period of one to 2 weeks of intensive physiotherapy treatment after MUA is essential to prevent recurrence of restrictions. Therefore, we advocate a more aggressive rehabilitation with intensive stretching and range of motion exercises in the first weeks after MUA to preserve the obtained range of motion.

In the current situation in our hospital, a variability in the threshold to decide for MUA between the different individual orthopaedic surgeons in the treatment of FS was noticed. This variability was also demonstrated in a survey among Dutch and Belgian orthopaedic surgeons [26]. In addition, we found that MUA was carried out most frequently at our hospital. In anticipation of the current protocol for an RCT, we reviewed our own results after manipulation in a retrospective cohort study. In 2 years, 89 patients were treated by manipulation for a FS. Eighty-five percent of the patients were satisfied with the procedure with good results. No complications were noticed. (T Kraal et al. *Acta orthopaedica Belgica* 2017, in press).

The objective of this trial is to evaluate the effectiveness of MUA followed by a PT program compared to a PT program alone in the treatment of patients with a stage two FS. We hypothesize that the course of the disease can be shortened with MUA with a quicker functional recovery and gain in range of motion and a subsequent faster return to work compared to physiotherapy treatment.

## Methods

### Study design

This trial is a prospective, single center, randomized controlled trial. The study is conducted the Amphia hospital Breda, one of the largest teaching hospitals in the Netherlands. Four shoulder specialists represent the Upper Limb Unit and will participate in the trial.

### Recruitment and consent

**Fig. 1:**
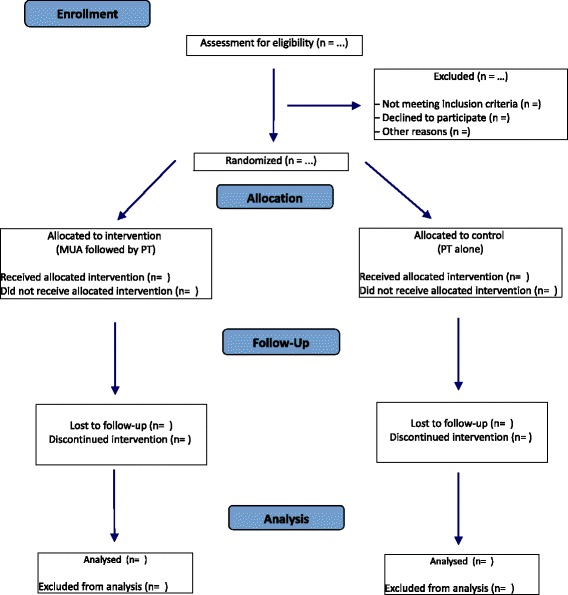
Participant flow diagram

All adult patients presenting to the outpatient with the clinical diagnosis of a FS in stage two will be invited to participate in the trial. A general history is acquired. The upper extremity is examined and range of motion is measured. Conventional radiographs (true anteroposterior in the scapular plane, internal rotation with 90 degrees of flexion in the elbow and the forearm in front of the abdomen, and in maximal external rotation) are made at baseline, to rule out other pathology such as osteoarthritis. The treating orthopaedic surgeon or a member of the study staff will introduce and explain the trial to the patient and address any further questions. The patient will receive a written information leaflet together with an informed consent form. After ample time to consider participation in the trial, patients return to the outpatient clinic. After receiving verbal and written consent, eligible patients will be randomized. A secure web based randomization program (CASTOR, https://www.castoredc.com/) is used for block randomization with differing block sizes and with a randomization allocation ratio of 1. This randomization schedule is only accessible for the research coordinator. Applicants will be allocated to either the MUA group or the PT group. Only the research coordinator (who is not a treating physician) will be authorized to use the randomization software module in CASTOR to allocate patients to their intervention group, hereby ensuring concealed allocation. A participant flow diagram is shown in Fig. 1. Blinding of patients is not possible. Range of motion measurements are done by a nurse practitioner, blinded for the intervention. Crossing over (from PT to MUA) is potentially possible because patients are allowed to quit participation in the trial as a personal choice. However, the results will be analysed based on the initial treatment allocation using the intention to treat (ITT) analysis (see ‘statistical analysis’ section for more details).

If patients visit the outpatient clinic with a stage one FS, they are not (yet) eligible for inclusion in the study. A standardized treatment regimen will be followed, as is the current usual care. They are given advice and education about the condition, the prognosis and the possible treatment options are discussed. An informative brochure and referral to a physiotherapist with instructions is given. An intra-articulair corticosteroid infiltration is discussed and directly administered if desired. Information about the trial is provided. Evaluation takes place after 3 months at the outpatient clinic and eligibility for inclusion in the study will be reassessed.

### Study population

This study focusses on patients with a clinical diagnosis of a stage two FS. This is defined as symptoms of pain and stiffness, predominantly in one shoulder, persisting ≥3 months, without preliminary trauma which led to an anatomic abnormality. Characteristically, the pain is most severe at the end of the range of motion. Pain must be diminished compared to the maximum amount of pain in stage one of the condition.

In order to be eligible to participate in this trial, patients must meet all of the following criteria:

- Age > 18 years and ≤70 years
- Restriction of passive motion in the glenohumeral joint of ≥30° in external rotation and at least a second plane of movement with ≥30°restriction (compared to the contra-lateral side)
- Unsuccessful conservative therapy within the previous 3 months. This is considered as insufficient improvement after an intra-articular corticosteroid infiltration and physiotherapy treatment during at least 6 weeks.

Patients with diabetes are eligible for participation in this trial.

If any of the following criteria will apply, patients will be excluded from participation:

- Numeric Pain Rating Scale at rest ≥7
- Onset of symptoms ≥1 year ago
- Osteoarthritis of the glenohumeral joint, Kellgren-Lawrence osteoarthritis grading scale ≥2
- Previous surgery to the shoulder
- Systemic inflammatory joint disease
- Evidence of a complete rotator cuff tear on physical examination, ultrasound images or MRI
- Neurological disorders of the upper limb
- Therapeutic anticoagulation which can not be interrupted without bridging therapy
- Other known shoulder pathology such as infection or tumor
- Contra-indication to corticosteroid injection, allergy to contrast or local anaesthetic
- Inability to give informed consent and fill out questionnaires

### Intervention

Patients assigned to the MUA group will be scheduled for the intervention within approximately 2–6 weeks (generally within 2 weeks). MUA is performed by one orthopaedic surgeon (RB) at the recovery room under single shot interscalene brachial plexus block. The interscalene block is administered by the anesthesiologist using ultrasound guidance. Levobupivacaine 0.375% is used, and a ‘soak time’ of approximately 45 min is pursued. If necessary or desired by the patient, general anesthesia can be added. The scapula is indirectly stabilized by the supine position, a short lever arm and 90 degrees of elbow flexion is used to prevent fractures and brachial plexus traction injuries. The glenohumeral joint is forced through a full range of motion in a strict pattern: anteflexion - > abduction, external rotation in 90 degrees’ abduction- > internal rotation in 90 degrees’ abduction - > horizontal adduction with dorsal compression and external rotation in neutral. A recognizable tearing sound is typically present when dealing with a FS. This sequence can be repeated until full range of motion is acquired. Postoperative physiotherapy is started directly on the same day (within 4 h after MUA) to maintain the acquired full range of motion. People stay at the orthopaedic ward for one night. The first week after MUA, patients have to visit a physiotherapist on a daily basis. The physiotherapy treatment is individualized in the need of the particular patient and it’s possibilities in ROM and dysfunction after a long period of stiffness. Therapy will exist of mobilizations in all end ranges known in arthrokinematics of the shoulder which are the same used by the orthopedic surgeon during MUA. Mobilizations are applied in (Maitland) grade 3, 4 or even 5 if necessary. This means that end feel is reached even if painful. The target is to reach the same end range as reached by the orthopedic surgeon after MUA, or the best possible after anesthetics are worked out. It is continuously tried to be within the pain area of NRS 0 to 5 or even up to NRS 7 for a short period of time, but only then when the pain vanishes within one or 2 h after therapy. The goal is to give the maximum of stimulus which the patient can handle. Therefore, the frequency of therapy is high, but the period of inflammation after manipulation is respected. Patients are given a home exercise program to maintain ROM which they have to imbed in their daily activities. The exercises will mainly concern stretching in different angles with a total end range time of at least 2 min. After 2 weeks, if ROM is maintained, a general exercise program is applied to regain function of cuff and scapular muscles (using elastic exercise bands or halters) with the goal to return to normal shoulder girdle function.

**Fig. 2:**
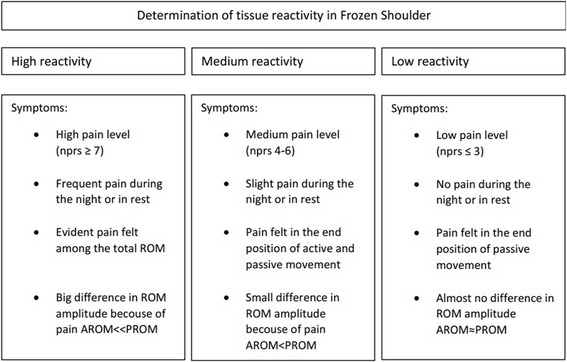
Determination of tissue reactivity in Frozen Shoulder

**Fig. 3:**
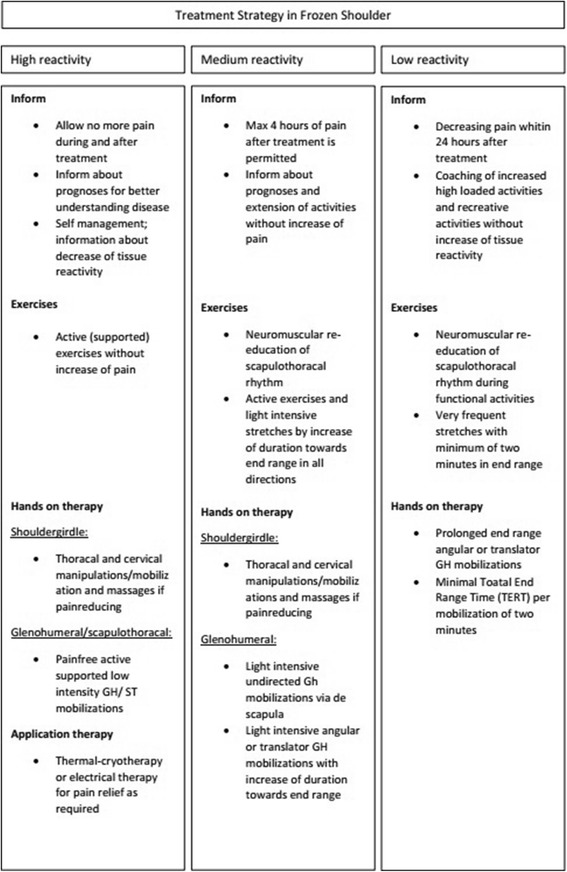
Treatment strategy guideline based on tissue reactivity

Patients allocated to the PT group are given advice and education about the natural course of the disease. A corticosteroid injection in the glenohumeral joint of kenacort 40 mg (1 ml) and chirocaine (4 ml) is given within the first 3 months of the condition, thus this will be done before inclusion in the study is possible. When the pain is not sufficiently diminished, this can be repeated. An advice for physiotherapy is given with a written protocol to hand out to their physical therapist based on the recent guideline of the Dutch Shoulder Network for the treatment of frozen shoulders (Fig. 2). This guideline uses a categorization in “tissue reactivity” with parameters of pain and ROM [16] that guides the treatment intensity and strategy (Fig. 3). A variety of treatments is used including, passive stretching, mobilisation techniques, active scapulothoracic exercises and cuff exercises. The Duration of physiotherapy treatment depends on the individual progression.

### Outcome measures

The primary objective of this study is to evaluate the difference in functional outcome after treatment of a FS with or without MUA, measured by the SPADI at 1 month compared to baseline. The SPADI is a self-reported questionnaire, with 13 questions responded on a ten-point scale divided in two domains: pain (5 items) and disability (8 items). A total SPADI score is calculated by summing up all 13 items and dividing by 130 (the maximum score) times 100. This leads to a score between 0 (best) and 100 (worst) [27]. The SPADI has been translated and validated in Dutch [28, 29].

Secondary outcome measures consist of;

- Oxford Shoulder Score (OSS), which reflects both function and pain of the shoulder. The OSS is a patient reported questionnaire, which consist of 12 questions related to pain and function of the arm in daily life. Items are scored on a zero to four point scale. This leads to an OSS score between 0 (worst) and 48 (best) [30].
- Shoulder pain at rest, and during activity; Pain level will be determined using the The Numeric Pain Rating Scale (NPRS). The NPRS is a validated 11-point score to assess pain, which represents a valid measure of pain with a good construct validity. The NPRS ranges from zero to ten, in which zero expresses no pain and ten expresses the worst pain possible.
- Health related quality of life, determined using the three level EuroQol five-dimensional questionnaire (EQ-5D 3-L). The EQ-5D is a five question standardised questionnaire scoring on five domains: mobility, self-care, usual activities, pain/discomfort, and anxiety/depression. It also includes a VAS self rating health scale on which patients rate their health state (0 is worst imaginable health and 100 is best imaginable health) [31]
- Passive Range of Motion (ROM) is measured by a goniometer. Forward flexion and abduction in the standing position, external rotation measured with the arm held at the side and the elbow in 90° flexion. Internal rotation is estimated to which height the patient can reach on his back, appointed to the highest vertebral level of the wrist.
- The ability to work is evaluated by two questionnaires. The WORQ-UP is a patient reported questionnaire with 17 items of common physical tasks at work, scored on a five point scale [32]. Single item work ability Index is a single question whereby patients rate their ability to perform physical tasks at work on a ten point scale. Zero indicates no ability to perform work with any physical task at all. Ten indicates the best period in life to perform physical tasks at work [33–35]. Absenteeism at work is evaluated with a single question where patients register the amount of days absent at work past month due to complaints of the shoulder.
- Duration of symptoms is determined. Patients are asked to estimate the duration of symptoms in weeks from MUA or allocation to the PT group until almost full recovery.
- Two anchor questions will be asked regarding the change that is experienced since the start of treatment considering pain and daily functioning. This is reported on a seven-point scale. These questions are based on an advice to use them from the division shoulder and elbow from the Dutch Orthopaedic Society.
- Quantity of physiotherapy treatment sessions
- Usage of analgesics (acetaminophen, NSAID’s or opioids)
- Number of repeated corticosteroid infiltrations
- Number of complications (infection, fracture, dislocation, neurovascular compromise, subsequent or intervention) will be registered and evaluated.

Passive range of motion is the only blinded outcome measure. All other outcomes are assessed unblinded or self reported.

### Study procedures

**Fig. 4:**
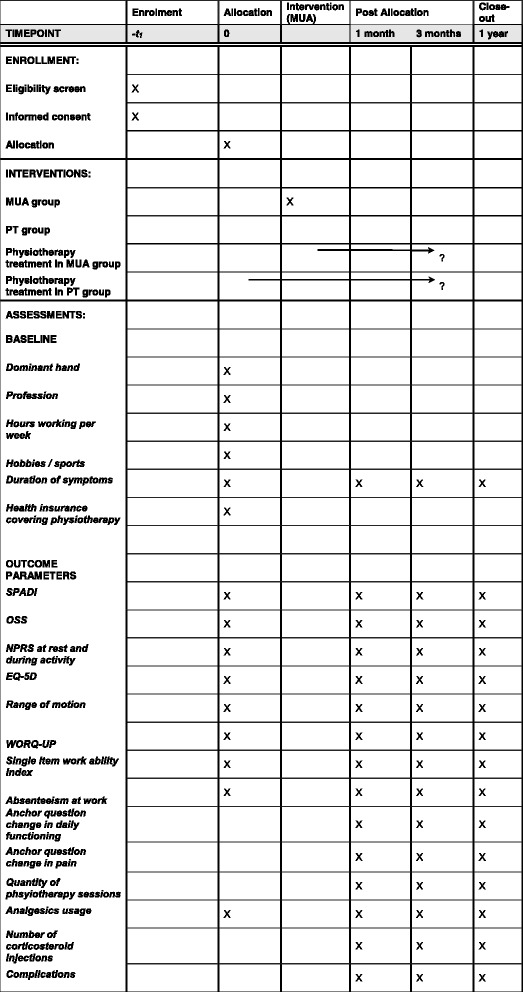
Schedule of enrollment, intervention and assessments

At 1, 3 months and 1 year, relevant outcome data are collected through clinical evaluation performed by the trained nurse practitioner, an orthopaedic surgeon, or resident in orthopaedic surgery. The range of motion is measured by a trained nurse practitioner who is blinded for the intervention. In the MUA group, the first follow up time is 1 month after the intervention. In the PT group, the first follow up time is 1 month after allocation. The schedule of enrolment, interventions, and assessments is shown in Fig. 4. The duration of the physiotherapy program in both groups is variable and depends on the individual result and desire of the patients. It is up to the patient to discuss this with their individual physiotherapist.

### Sample size calculation

The SPADI is the primary outcome parameter. The sample size calculation is based on the ability to detect a difference between treatment groups of ≥13 points in the total SPADI scores. This is based on the study of Schmitt which describes a minimal clinical important difference (MCID) of 13 [36]. The study of Carette shows a standard deviation of 17 on the SPADI [37]. Based on these parameters, we calculated a sample size of 41 subjects per group with a power of 90%, alpha 0.05 and a 10% drop out rate.

### Statistical analysis

All data will be analysed in an encoded fashion. We will use CASTOR (https://www.castoredc.com/), an online data-management program, designed for medical research purposes. The patient’s demographic characteristics, EQ-5D score, range of motion at baseline, duration of symptoms before treatment starts and diabetes mellitus will be summarized and compared between groups. Also the distribution of all patient’s outcome variables will be summarized by treatment group and by time. The summaries will consist of the following descriptive statistics: number of patients involved, mean and standard deviation (or median and (inter quartile range when appropriate) for continuous variables and relative frequencies (percentages) for categorical variables. We will report the number of participants (denominator) included in each analysis and the Intention To Treat principle will be used with respect to group assignment. So, the final results of the patients will be analysed in the group to which they were allocated at the start of the study.”

The SPADI-measurements will be analysed using linear mixed modelling. The restricted maximum likelihood technique will be used for estimating the model parameters. The independent variables are time (3 levels: 1, 3 and 12 months) and treatment (2 levels), as well as their interaction. The following baseline covariates will also enter the model: SPADI at baseline, diabetes and duration of symptoms before intervention.

Primary efficacy measure is the treatment effect (MUA vs. PT) on total SPADI score after 1 month. This effect is estimated as contrast on the coefficients of the linear mixed model including the treatment-by-time interaction as mentioned above. Missing values in patients with incomplete observations will be appropriately dealt with by using the restricted maximum likelihood technique. Secondary efficacy measures are the treatment effects after 3 and 12 months and an average treatment effect over time obtained by deleting the treatment-by-time interaction. In addition, this interaction will be tested as part of the secondary efficacy analysis. Other secondary continuous outcome variables, such as OSS, NPRS, EQ-5D, WORQ-UP and ROM, will be analysed similarly to SPADI, with the baseline measurement of the outcome variable at hand as covariate. When appropriate, the outcome variable will be transformed so as to obtain normally distributed residuals.

Complications are counted by type and in total and will be analysed using Poisson regression analysis with a correction for overdispersion when appropriate. Treatment effects on complication rates will be expressed as MUA to PT rate ratios. Safety will be assessed by identifying and summarising adverse events collected throughout the study.

All estimated treatment effects will be accompanied by 95% confidence intervals and *p*-values.

Analysis will be performed by use of SPSS statistical package (IBM, version 18.0; SPSS, Chicago, Illinois).

### Ethical considerations

There is insufficient evidence in the current literature for either one of the treatment allocations in this study. Both treatment strategies (MUA and PT) are regularly applied for a stage two FS in our hospital for many years. The intervention MUA will be performed by one orthopedic surgeon (RB) who has an extensive experience with this procedure. The treatment protocol of both treatment groups are kept close to the current routine care for patients with a similar condition not enrolled in the study. Patients will be exposed to radiation from conventional radiographs before inclusion of the study. This is part of routine clinical care and represents no increased risk. Patients may experience the questionnaires as inconvenient, but we consider this a minor inconvenience as they will take approximately 10 min to complete.

The motivation for the study is a potential benefit to all patients with a stage two FS, as we increase our knowledge on optimal treatment strategy for this condition.

### Monitoring and quality assurance

The study was registered by the CCMO (National Central Committee of human bound research) under the number NL.56143.101.16 and registered in the Dutch Trial Register under the number NTR6182. The study protocol has been approved by the medical ethical committee TWOR (toetsingscommissie wetenschappelijk onderzoek rotterdam e.o.) Maasstad hospital Rotterdam and local feasibility was tested by the AMOA (adviescommissie mensgebonden onderzoek amphia) committee of the Amphia hospital Breda. Independent trial oversight was not deemed necessary by the medical ethical committee, because both treatments are already used for a long period in our hospital. For this reason, the patients are not expected to be at risk by participating in the current study.

All informed consent forms will be filed in a locked cabinet in the research office. Results of physical examination and questionnaires will be collected digitally and stored on a password-protected, secured server to which only study staff will have access.

All investigators will be responsible for reporting adverse events to the coordinating investigator. The coordinating investigator will report any adverse events to the ethical committee in accordance with the ethical committee adverse event reporting procedures. The coordinating investigator and the principal investigator are responsible for adherence to all ethical committee rules and guidelines and for the accuracy and completeness of all forms, entries and informed consent.

## Discussion

There is no consensus in the literature which patients with a FS will benefit most from MUA [11, 15, 17]. MUA is considered as an option when conservative treatment fails. However, the optimal timing of MUA is unknown [12, 38]. Furthermore, timing between the onset of symptoms and MUA can be a crucial parameter when the effectiveness of MUA is evaluated. In a condition that is mainly self-limiting, shortening of the duration of symptoms is probably more interesting than the end result at long term. Theoretically, the biggest advantage of manipulation is obtained when manipulation is done early. It could be suggested that early manipulation could lead to over-treatment in patients with a mild and natural course of the disease. Even more, early manipulation in stage one (the painful inflammatory phase) is sometimes considered to be counterproductive and can lead to recurrence of symptoms [12]. On the other hand, a high threshold for MUA, or late intervention, can lead to an unnecessarily long duration of complaints. In a retrospective study, Vastamäki et al. considered between 6 and 9 months after the onset of symptoms as the most optimal period for manipulation [12]. However, only a general comparison between group A (between 6 and 9 months) and group B (the others, including before 6 months and after 9 months) was presented. In our opinion, this is not convincing evidence to draw firm conclusions about the optimal timing. Although clear cut-off values between different stages of a FS are lacking, we decided to define in- and exclusion criteria as described above to select patients with a FS in stage two. The exclusion criteria NRS ≥ 7 is debatable because the lack of cut-off values in the literature. We added the important parameter that pain must be diminished compared to the maximum pain in stage one. Hereby, we try to prevent over treatment and recurrence after too early manipulation. Furthermore, with this study protocol, unnecessarily long duration of symptoms are potentially avoided. With the results of this study, we aim to increase our knowledge about the efficacy of MUA compared to physiotherapy treatment. We aim to solve a part of the uncertainty of the indication of MUA, and the safety of MUA is critically assessed.

## Abbreviations

FS: Frozen shoulder
MUA: Manipulation under anesthesia
PT: Physiotherapy treatment
